# Receptiveness to participating in cannabis research in pregnancy: a survey study at The Ottawa Hospital

**DOI:** 10.12688/f1000research.51947.1

**Published:** 2021-05-24

**Authors:** Kira Bombay, Malia SQ Murphy, Kathryn M Denize, Christina Cantin, Amy McGee, Ruth Rennicks White, Shi Wu Wen, Mark C Walker, Daniel J Corsi

**Affiliations:** 1 OMNI Research Group, Ottawa General Hospital Research Institute, Ottawa, Ontario, K1H 8L6, Canada; 2 Champlain maternal Newborn Regional Program, Ottawa, Ontario, K1G 4J8, Canada; 3 School of Nursing, Queen's University, Kingston, Kingston, Ontario, K7L 3N6, Canada; 4 Department of Midwifery, Ottawa Hospital, Ottawa, Ontario, K1Y 4E9, Canada; 5 Department of Obstetrics and Gynecology, University of Ottawa, Ottawa, Ontario, K1H 8L6, Canada; 6 School of Epidemiology and Public Health, University of Ottawa, Ottawa, Ontario, K1G 5Z3, Canada; 7 Department of Obstetrics, Gynecology & Newborn Care, Ottawa Hospital, Ottawa, Ontario, K1H 7W9, Canada; 8 BORN Ontario, Children's Hospital of Eastern Ontario, Ottawa, Ontario, K1H 8L1, Canada; 9 Children's Hospital of Eastern Ontario, Ottawa, Ontario, K1H 5B2, Canada

**Keywords:** Pregnancy, cannabis, participation, willingness, motivators, birth cohort

## Abstract

**Background:** The prevalence of cannabis use among pregnant individuals in Canada is increasing. In the design of new cohort studies to evaluate the patterns and outcomes of cannabis use in pregnancy, consideration must be given to the factors influencing participation, data sharing, and contribution of biological samples. Our objective was to assess the willingness of pregnant individuals to participate in prospective research during pregnancy.

**Methods:** We surveyed pregnant individuals receiving obstetrical care through The Ottawa Hospital in Ottawa, Canada. The survey consisted of 23 dichotomous (yes/no), multiple-choice, Likert scale, and open-ended questions. Individuals were provided with a hypothetical research scenario and asked to report on the likelihood of their participation, use and storage of personal health information and contribution of maternal and newborn samples. Individuals provided motivating and deterring factors related to research participation. Descriptive statistics included frequencies (n) and percentages (%) for categorical variables. Continuous variables were described using means and standard deviations.

**Results:** A total of 84 survey responses were collected. The mean age of respondents was 32.6(±5.3) years. Respondents were predominantly Caucasian (79%), college/university educated (85%) with a household income of ≥$100,000 (64%). There was a high degree of willingness to participate in prospective research by sharing data and biological samples. The most commonly cited motivating and deterring factors for participating in future research were a desire to contribute to science and health information (79%) and fear of privacy invasion (17%), respectively.

**Conclusions:** Pregnant individuals receiving care at The Ottawa Hospital are willing to participate in prospective research studies, including those related to cannabis use. Survey respondents were predominantly of higher socioeconomic status, and few individuals reported cannabis use during pregnancy. Future studies should accommodate multiple recruitment strategies and flexible study designs to encourage enrollment from and retention across diverse sociodemographic communities.

## Introduction

The prevalence of cannabis use among pregnant individuals in Canada is increasing. In Ontario, self-reported cannabis use in pregnancy increased from 1.2% in 2012 to 1.8% in 2017, a relative increase of 61%. ^1^ These increases are particularly notable among individuals 15-24 years old, among whom self-reported cannabis use in pregnancy has increased to more than 6% in recent years. ^2^ Such findings are a significant public health concern given accumulating evidence suggesting an association between cannabis use in pregnancy and risk of stillbirth, small for gestational age, lower birth weight, and increased admission to neonatal intensive care compared with infants without cannabis exposure. ^1, 3– 5^ Longer-term impacts on child neurodevelopment and mental health have also been reported. ^6– 8^

Although information on the frequency, timing, dose and intake method of cannabis use is essential for inferring its health impacts on exposed individuals, population-based data on cannabis use in pregnancy are frequently lacking this level of information. Reports based on prospective data collection are few and frequently limited by small sample sizes, reliance on self-reported data and other methodological challenges. ^9, 10^ Cannabis use in pregnancy is associated with negative social stigma and perceived consequences of reporting drug use in pregnancy, such as fear of losing children and fear of prejudicial treatment, ^11^ may dissuade individuals from participating in research or accurately disclosing their drug-related habits. Indeed, previous work comparing self-reported data against urine toxicology screens has demonstrated that individuals are likely to underreport substance use in pregnancy, including cannabis use. ^12, 13^ There is a pressing need to generate current, robust data on cannabis use in the Canadian obstetrical population, to improve our understanding of the trends, determinants, and outcomes of cannabis use in the post-legalization setting.

The requirements of longitudinal research are particularly burdensome for the obstetrical population, who are already faced with accommodating frequent health care appointments in addition to professional and personal obligations. In the design and enrolment of participants into large-scale birth cohorts, consideration must be given to the factors influencing participation, data sharing and contribution of biological samples. We surveyed pregnant individuals to determine their receptiveness to enrolling in a hypothetical birth cohort designed to evaluate the prevalence and safety of cannabis use in pregnancy. Our objective was to assess factors influencing pregnant individuals’ motivations and receptiveness to research participation.

## Methods

### Study design, setting and population

This was a cross-sectional survey study of individuals receiving obstetrical care through The Ottawa Hospital in Ottawa, Ontario. Ottawa is the second largest city in Ontario and the fourth largest in Canada. The census metropolitan area of Ottawa-Gatineau has a population of 1.3 million. ^14^ The Ottawa Hospital is a Level 3 maternity care hospital with antenatal clinics and birthing units at two hospital campuses that provide obstetrical and neonatal care to the Ottawa-Gatineau region. During registration at The Ottawa Hospital, all patients are asked if they would like to give their permission to be contacted for future research studies through the institutional *Permission to Contact* program. ^15^ For this study, individuals were eligible to participate if they gave permission to be contacted for research purposes, were 18 years of age or older, pregnant, and receiving care through The Ottawa Hospital, or if they had recently delivered and were still in hospital. Pregnant individuals receiving care at St. Mary’s Home, a satellite clinic of The Ottawa Hospital available to disadvantaged pregnant youth where clients under 18 are considered emancipated minors, were also eligible. This patient population includes youth experiencing, or at-risk for, homelessness, addictions, family and intimate partner violence, and other challenges.

In January 2020, we mailed 656 survey packages. Mailed packages included a participant information sheet, survey, pre-paid return envelopes, and a recruitment poster with a link to an online version of the survey. In this way, individuals receiving the mailed packages could complete the survey on paper or online. Recruitment posters advertising the online survey were placed in clinic waiting areas of The Ottawa Hospital and St. Mary’s Home, and postpartum wards. Using an iPad to participate in the survey was also an option for those completing the survey at St. Mary's Home. Paper-based surveys were also available alongside a secure drop-box for individuals to deposit their responses. All materials were available in both English and French. Survey responses were anonymous.

### Survey

The survey consisted of 23 dichotomous (yes, no), multiple-choice, Likert scale, and open-ended questions. Respondents self-reported demographic and socioeconomic characteristics. These included age (years), race/ethnicity (Indigenous, White, Chinese, Black, Filipino, Latin American, Arab, Southeast Asian, West Asian, Korean, Japanese, Other), level of education (grade school, high school, college/university attended but not completed, college/university completed), household income (less than $25,000, $25,000-$49,999, $50,000-74,999, $75,000-$99,999, more than $100,00), marital status (married or common law; separated or divorced; single, never married; widow or widower) and obstetrical history (gravidity, parity).

Questions specific to cannabis-use covered topics including current knowledge regarding how cannabis use can affect pregnancy and postpartum health (e.g. “Do you think that using cannabis during pregnancy affects: a) your pregnancy health, b) your baby’s health, c) your breastmilk, or d) I don’t know”); and participant use of alcohol, tobacco, cannabis, or other substances (any time before pregnancy, in the current pregnancy, never). Individuals were asked if their healthcare provider had discussed the topic of cannabis use in pregnancy with them (yes, no) and to identify their primary sources for information on cannabis (friends and family; health care professionals; government websites; media, general internet searches; cannabis producers, distributors or retail providers; other).

**Figure 1.**
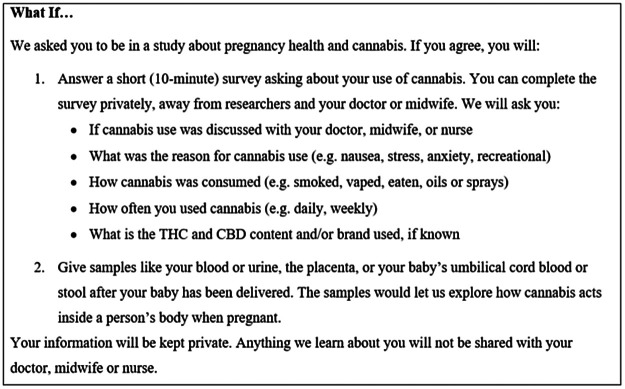
Hypothetical Research Study Prompt Given to Participants.

The survey included a description of a hypothetical research study in which researchers sought to collect data and biological samples during pregnancy to explore cannabis use patterns, habits and preferences ( Figure 1). After reading the description, individuals were asked how likely they would be to participate in the hypothetical research study if invited, if their partner would support their participation, and if their partner would participate themselves (answer choices: very likely, likely, unlikely and very unlikely). Questions also covered individuals’ willingness to self-report cannabis use in pregnancy and provide different types of biological samples, such as urine, blood, saliva, cord blood, newborn stool, placental tissue, and breastmilk once or multiple times throughout pregnancy. Individuals were also asked about their willingness to consent to the storage of biological samples for future use and have their data linked to their health records. Using a pre-specified, multi-select list, motivating and deterring factors for participating in cannabis-related research were captured. Individuals were asked to identify factors that would motivate them to participate the hypothetical research study described (i.e., contributing to science and health information, learning more about cannabis and pregnancy health, helping future patients, getting paid money, other), and identify any concerns they would have about sharing information related to cannabis use with researchers (i.e., being judged, feeling embarrassed, feeling guilty, being reported to child protective services, privacy concerns, changes to the healthcare they receive, no fears, other).

### Statistical analysis

Descriptive statistics included frequencies (n) and percentages (%) for categorical variables. Continuous variables were described using means and standard deviations, and minimum and maximum values when appropriate. Likert-scale survey responses “very likely” and “likely” were combined, as were responses of “very unlikely” and “unlikely”.

***Ethics***

This research was approved by the Ottawa Health Sciences Network (OHSN) Research Ethics Board (20190529-01H). At the start of each survey, participants were provided with information outlining the purpose and nature of the survey study. Participants were informed that the survey responses were anonymously collected, and how the data would be used (including for publication), stored, and destroyed. Participants were informed that their participation was completely voluntary and that by completing the survey, their consent to participate was implied. Thus, informed consent was obtained from all participants included in this paper. Research and ethics contacts were provided to the participants in case they had any questions or concerns about the study, or about data collection or management. The OHSN Research Ethics Board reviewed and approved all study documents, including the consent process.

***Consent for publication**:* Informed consent was obtained from all subjects.

## Results

### Participant characteristics

A total of 84 completed surveys were collected between January and March 2020. Of the 656 surveys sent via mail, 16 (2.4%) were returned due to invalid or change of address, meaning 640 surveys were delivered successfully. Seventy-five (89.3%) individuals reported that they had received the survey in the mail; a response rate of 11.7% (75/640). The remaining nine (10.7%) survey responses were completed by individuals who indicated that they had completed the survey after seeing recruitment posters in-hospital or because they had been recruited by research staff. Five patients were recruited from the St. Mary’s satellite clinic. The mean age of respondents was 32.6 (±5.3) years. Sixty-six (78.6%) survey participants were Caucasian, 71 (84.6%) completed college or university degrees, 78 (92.9%) were married or common-law, and 54 (64.3%) had a household income of over $100,000 ( Table 1).

**Table 1.** Participant Characteristics.

**Characteristic**	**n(%) or mean (SD)**
**Total number of individuals**	**84 (100 %)**
**Age, years, Mean (SD) (min/max)**	
Mean/Average (SD)	32.6 (±5.3) (20/43)
**Race/ethnicity** ^**a**^	
White/Caucasian	66 (78.6 %)
Black	5 (6.0 %)
Other	11 (13.1 %)
Missing	2 (2.4 %)
**Education**	
High school	7 (8.3 %)
College/university (attended but not yet completed)	3 (3.6 %)
College/university (completed)	71 (84.6 %)
Missing	3 (3.6 %)
**Household income**	
≤$50,000	5 (6.0 %)
$50,000 - $74,999	11 (13.1 %)
$75,000 - $99,999	9 (10.7 %)
≥$100,000	54 (64.3 %)
Missing	5 (6.0 %)
**Marital status**	
Married or common law	78 (92.8 %)
Single, never married	4 (4.8 %)
Missing	2 (2.4 %)
**Primary language spoken at home**	
English	57 (67.9 %)
French	13 (15.5 %)
Other	12 (14.3 %)
Missing	2 (2.4 %)
**Alcohol use history** ^**a**^	
Before pregnancy	63 (75 %)
During pregnancy	9 (10.7 %)
Never	10 (11.9 %)
Missing	2 (2.4 %)
**Tobacco use history** ^**a**^	
Before pregnancy	20 (23.8 %)
During pregnancy	0
Never	62 (73.8 %)
Missing	2 (2.4 %)
**Cannabis use history** ^**a**^	
Before pregnancy	39 (46.4 %)
During pregnancy	2 (2.4 %)
Never	38 (45.2 %)
Missing	5 (6.0 %)
**Other drugs use history (e.g. non-prescription opioids, cocaine, heroin, crystal meth)** ^**a**^	
Before pregnancy	6 (7.1 %)
During pregnancy	0
Never	70 (83.3 %)
Missing	8 (9.5 %)
**Gravidity**	
1	35 (41.7 %)
2	15 (17.9 %)
≥3	31 (36.9 %)
Missing	3 (3.6 %)
**Parity**	
0	42 (50.0 %)
≥1	39 (46.4 %)
Missing	3 (3.6 %)

^a^ This was a multi-select field, individuals could select multiple options. Percentages do not add to 100%. SD, standard deviation. Data are provided as N(%), unless otherwise indicated. Not all individuals provided complete information, number of individuals with non-missing values noted for each item. Percentages may not add to 100 due to rounding.

Sixty-three (75.0%) individuals reported previous alcohol consumption, 20 (23.8%) indicated prior tobacco use, 39 (46.4%) reported prior cannabis use and six (7.1%) reported using other non-prescription drug use at some point before their pregnancy. When asked to indicate substance use in their current pregnancies, nine (10.7%) individuals reported alcohol use, and two (2.4%) reported cannabis use. None of the surveyed participants reported tobacco or other drug use in their current pregnancies.

### Information sources and perceptions on substance use in pregnancy

A total of 67 (79.8%) individuals reported that their healthcare provider had not yet discussed cannabis use during pregnancy with them ( Table 2). The three most common sources of information on cannabis used by individuals were family and friends, media (e.g., news, magazines, and social media), and general internet searches (44.0%, 39.3% and 32.1 %, respectively). Most individuals believed that using cannabis during pregnancy would affect pregnancy health (62, 73.8%), their baby’s health (70, 83.3%), and their breastmilk (62, 73.8%). 14 (16.7%) individuals indicated that they did not know if cannabis use in pregnancy would affect pregnancy health, infant health or breastmilk.

**Table 2.** Information Sources and Perceptions on Substance Use in Pregnancy.

**Characteristic**	**Total Number of Responding N (%)**
**Total Number of Respondents**	**84 (100 %)**
**Individuals who had a discussion about cannabis use in pregnancy with a healthcare provider**	
Yes	14 (16.7 %)
No	67 (79.8 %)
Missing	3 (3.6 %)
**Main sources of information on cannabis among individuals** ^**a**^	
Friends and family	37 (44.0 %)
Government websites	15 (17.9 %)
General internet searches	27 (32.1 %)
Health care professionals	15 (17.9 %)
Media (news, magazines, social media)	33 (39.3 %)
Producers, distributors or retail providers	1 (1.2 %)
I have not looked up information on cannabis	25 (29.8 %)
Other	7 (8.3 %)
Missing	2 (2.4 %)
**Participant perceptions about the extent of the effects of cannabis use during pregnancy** ^**a**^	
Affects their pregnancy health	62 (73.8 %)
Affects their baby’s health	70 (83.3 %)
Affects their breastmilk	62 (73.8 %)
Don’t know	14 (16.7 %)

^a^ This was a multi-select field, individuals could select multiple options. Percentages do not add to 100%.

### Willingness to participate in a hypothetical research study

**Figure 2.**
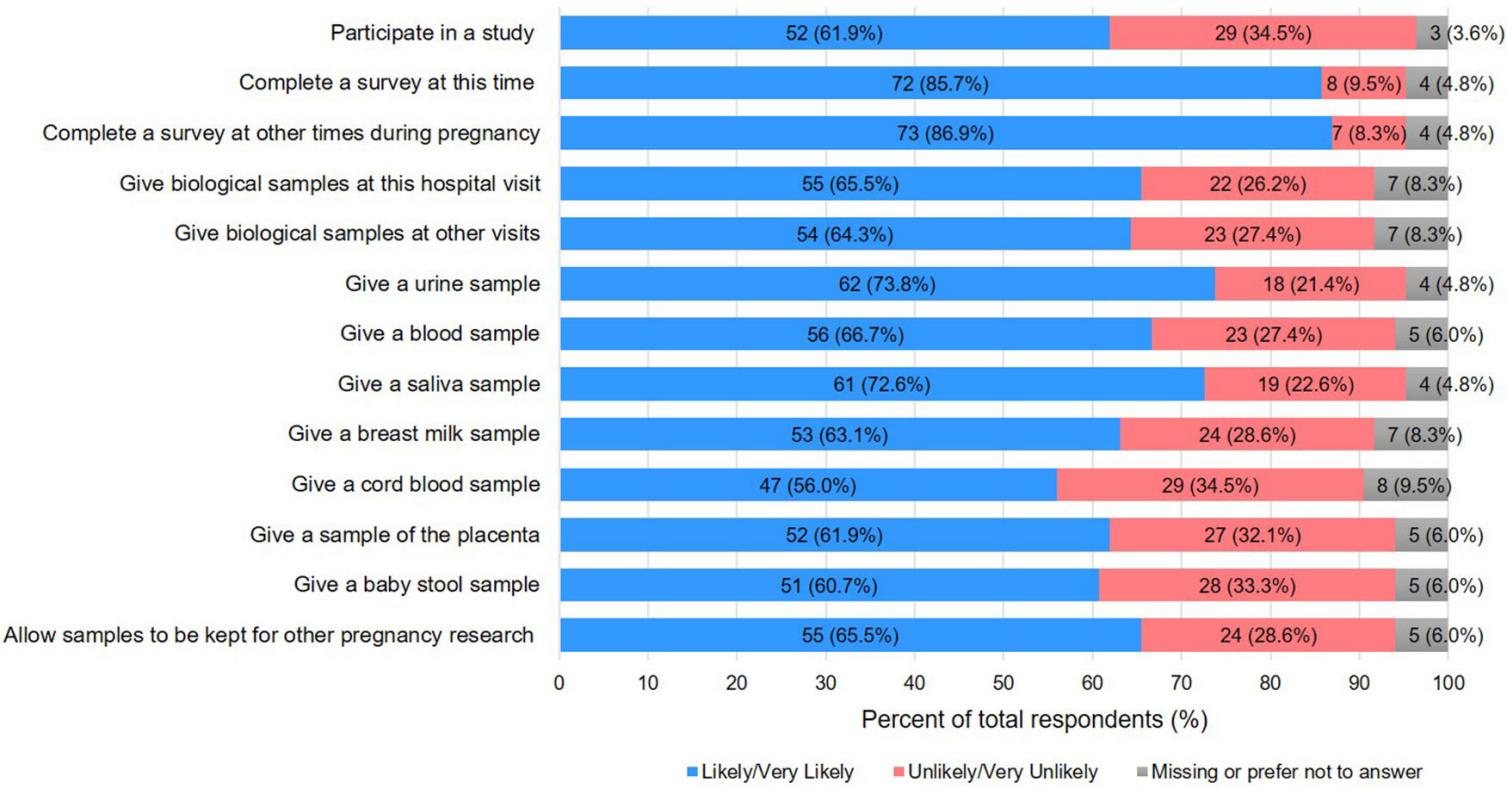
Willingness to Participate in Hypothetical Cannabis Research Study.

52 (61.9%) individuals reported that they would be willing to participate in the hypothetical research study ( Figure 2). 72 (85.7%) individuals said they would be likely to participate if asked to complete a survey now, and 73 (86.9%) said they would be likely to participate in doing so at other times later in their pregnancy. The majority of individuals were willing to give biological samples at the time they completed the survey or at subsequent hospital visits (55 (65.5 %) and 54 (64.3%), respectively). A higher proportion of individuals indicated that they would be willing to provide urine (62 (73.8%)) or saliva samples (61 (72.6%)) than any other sample type suggested by the hypothetical study. Individuals were least willing to consider providing placental tissue, cord blood, and newborn stool samples for the hypothetical study (>30% responding unlikely/very unlikely for each sample). Over half of the individuals reported being open to consenting to the storage and use of biological specimens for other pregnancy research (55 (65.5%)). 60 (71.4%) believed that their partners would be supportive of their research participation, though fewer (44(52.4%)) believed that their partners would also agree to be in the hypothetical research study.

### Motivating and deterring factors for participating in research

The most commonly cited motivating factors for participating in research was a desire to contribute to science and health information (66 (78.6%)), and the potential for helping future patients (49 (58.3%)) ( Table 3). Three (3.6%) respondents answered that there were no possible motivating factors to participate in cannabis-related research. Approximately two-thirds of individuals indicated that they had no fears for participating in this type of research (53 (63.1%)). The most commonly reported deterring factors included worries about maintaining information privacy (14 (16.7%)) and being reported to Child Protective Services (if they reported using cannabis in pregnancy) (10 (11.9%)).

**Table 3.** Motivating and Deterring Factors for Participating in Cannabis-related Research.

	**Total Number of Responding N (%)**
**Total Number of Responding**	**84 (100 %)**
**Motivating Factors** ^**a**^	
Contributing to science and health information	66 (78.6 %)
Learning more about cannabis and pregnancy health	28 (33.3 %)
Helping future patients	49 (58.3 %)
Getting paid money	20 (23.8 %)
No possible motivating factors	3 (3.6 %)
Other	2 (2.4 %)
**Deterring Factors** ^**a**^	
Being judged	8 (9.5 %)
Feeling embarrassed	3 (3.6 %)
Feeling guilty	9 (10.7 %)
Being reported to Child Protective Services	10 (11.9 %)
My privacy (i.e. that the researchers would tell others)	14 (16.7 %)
Changes to the healthcare I receive	7 (8.3 %)
I do not have any fears	54 (64.3 %)
Other	9 (10.7 %)
Missing	3 (3.6 %)

^a^ This was a multi-select field, individuals could select multiple options. Percentages do not add to 100%.

## Discussion

In this cross-sectional survey of 84 pregnant individuals living in Ottawa, Ontario, we demonstrate that pregnant individuals are receptive to participating in prospective research studies, including those related to cannabis use in pregnancy. A majority of individuals were willing to complete surveys, provide biological samples and consent to having their information and samples stored for future use. Nearly three-quarters of individuals believed that their partners would support their research participation, and half believed that their partners would also agree to participate. The primary motivating factors for research participation included contributing to science and health information and helping future patients.

Pregnancy and birth cohorts are rich sources of data on the role of early life exposures and biological mechanisms on the development of disorders and disease. ^17, 18^ In light of recent legislative changes regarding the availability and use of cannabis in Canada, prospective cohorts that collect robust clinical and biological data are needed to evaluate the impact of pre-conceptional and gestational cannabis exposures on maternal, infant, and pediatric outcomes in the Canadian population. Prospective cohorts evaluating the effects of prenatal cannabis use on pregnancy and child health are few, ^5, 19– 21^ and are frequently limited by small sample size, reliance on self-reported cannabis use data, and loss to follow-up. Given increases in potency and access to varied administration routes in the post-legalization era, new data using information collected from both self-reported use histories and objective measures of cannabis use from toxicology screening may elucidate epidemiological findings. ^10^

Motivators and deterrents for research participation are important factors to consider when designing birth cohorts, so that study designs and recruitment strategies can be optimized to improve enrollment, participation and retention. ^18^ Our findings confirm those of others that pregnant individuals are generally willing to participate in research and are motivated by altruism. ^22^ In a qualitative assessment of pregnant individuals’ attitudes toward multiple methods of data and biological data collection, Gatny and colleagues reported that key design features likely to encourage research participation among pregnant individuals included the following: emphasizing the contribution to science and improvements in care for others, clinical/societal importance of the study, and study designs that accommodated various degrees of participation. ^22^ The success of flexible enrollment models for birth cohorts has been demonstrated elsewhere, including the Ottawa obstetrical population. ^23^ Altruistic motivations for research participation have also been described among non-pregnant individuals who use substances. A recent study examining willingness to participate in longitudinal research among long-time medical cannabis users found that 85% of respondents would enroll if asked. ^24^ Motivating factors included a desire to help others and to further scientific information.

Our survey assessed a large breadth of hypothetical scenarios, motivating and deterring factors for research participation. Findings that pregnant individuals are generally receptive to contributing biological samples are promising. Given the need for objective measures of cannabis use/exposure in pregnancy, standardization of data collection tools assessing cannabis use would be helpful as the frequency, dose, format, and method (smoked, ingested, etc) of cannabis consumption will provide important information on the influence of cannabis exposure and pregnancy and child health outcomes. ^25– 27^ Our findings also revealed a lack of patient-provider discussion related to cannabis use in pregnancy, and participants cited friends, family, and the media as their primary sources of information on cannabis use. Despite having at least one prenatal visit with an obstetrical care provider at a tertiary care hospital, 80% of participants had not yet discussed cannabis use with their health care providers. This is consistent with recent data demonstrating that maternity care providers feel ill-equipped to provide evidence-based counselling to patients, ^28, 29^ and highlights a need for reliable, contemporary evidence on the safety of cannabis use in pregnancy. As new data emerge on the influence of paternal and second-hand cannabis exposures, insight from our study participants on the receptivity of their partners to participating in cannabis-use research may be useful to inform the design of clinical research studies on this topic.

The strengths of this study include broad dissemination to the obstetrical population receiving care at our tertiary care center and the anonymity of the survey. Selection bias may limit the generalizability of our findings to other obstetrical populations and maternity care settings. First, surveys were distributed to individuals who had previously consented to be contacted for research; thus respondents may have been more likely to respond favourably to the hypothetical research scenario proposed in the survey than the broader population. Second, the response rate from our mailed survey strategy was low (12%). Although response rates for survey studies vary widely by subject area, study population and data collection techniques, the response rates for this study are similar to other research surveys conducted in the perinatal population, among whom response rates typically vary between 10 and 20%. Although use of incentives and electronic and telephone reminders are known to increase responses rates for survey studies, we were unable to facilitate this in order to maintain anonymity of respondents. Third, our sample consisted of individuals from higher socioeconomic status background than that of the broader Ottawa population. A large proportion of our sample had received post-secondary education, were married and white, with a total household income of over ≥$100,000. These demographics are similar to other pregnancy cohorts initiated at The Ottawa Hospital, ^23, 30^, and positive associations between education and willingness to participate in research have been previously described. ^31– 34^ Finally, although we sought responses from individuals with diverse histories of substance use, the majority of survey respondents reported not using cannabis or other substances and this may have impacted the low concern among participants being reported to Child Protective Services. Individuals who use cannabis in pregnancy have been shown to have lowered perceptions of risk related to cannabis use, ^35^ and their perspectives on research participation, including motivating and deterring factors, should be considered when designing research studies on cannabis use in pregnancy. In contrast, alcohol was the most commonly reported substance used during pregnancy (11%). These findings are consistent with data from the Canadian Maternity Experiences Survey and the Canadian Perinatal Surveillance System’s 2013 Perinatal Health Indicators Report in which the proportions of women who reported drinking alcohol during pregnancy were 10.5% and 10.7%, respectively. ^36, 37^ Although we are unable to fully gauge differences between individuals who use cannabis in pregnancy and those who do not, our findings provide important insight into the motivating and deterring factors influencing participation in birth cohort studies.

## Conclusions

This study demonstrates that pregnant individuals are willing to participate in prospective research studies, including those related to cannabis use in pregnancy. Differences in preferences for participation such as completing surveys or providing samples may warrant a flexible model whereby individuals have the freedom to opt in an out of elements of their choosing. In the design of birth cohorts, multiple recruitment strategies are needed to encourage enrolment from and retention across diverse sociodemographic communities that are representative of the broader local population. Our findings are informative for researchers seeking to develop prospective studies in this area.

## Data availability

### Underlying data

Open Science Framework: Underlying data for ‘Receptiveness to participating in cannabis research in pregnancy: a survey study at The Ottawa Hospital’, DOI 10.17605/OSF.IO/UWDHP. ^16^

This project contains the following underlying data:

• Table 1. Cannabis Survey Results

Data are available under the terms of the Creative Commons Attribution 4.0 International license (CC BY 4.0).
