# Supplementary material for: Receptiveness to participating in cannabis research in pregnancy: a survey study at The Ottawa Hospital
Source: F1000Res. 2021 May 24;10:413. [Version 1] doi: 10.12688/f1000research.51947.1 (PMC11803439; doi:10.12688/f1000research.51947.1)
Supplement: Peer review history

# Reviewer report: Reviewer response for version 1

**DOI:** 10.5256/f1000research.55161.r327231

**Published:** 2024-12-30

**Authors:** Omayma Alshaarawy

**Affiliations:** 1 Michigan State University, East Lansing, Michigan, USA

The authors have developed and distributed a survey to assess the willingness of pregnant individuals to participate in cannabis research and provide biological samples. Overall, the paper is well written and easy to follow. The main limitation of the study is inherent in the sample, a relatively high SES and very low prevalence of prenatal cannabis use. Hence, selection bias is a real threat to validity here. Also, the study lacks specimen collection/analysis to verify responses related to cannabis and other drug use. The authors acknowledged these limitations and compared their results to the obstetric population in Ottawa. I believe the authors should compare the characteristics of their sample to what is known about individuals who use cannabis during pregnancy as they are the cornerstone of any prenatal cannabis use study.

Is the work clearly and accurately presented and does it cite the current literature?

Yes

If applicable, is the statistical analysis and its interpretation appropriate?

Yes

Are all the source data underlying the results available to ensure full reproducibility?

Yes

Is the study design appropriate and is the work technically sound?

Yes

Are the conclusions drawn adequately supported by the results?

Yes

Are sufficient details of methods and analysis provided to allow replication by others?

Yes

Reviewer Expertise:

Cannabis; Epidemiology; Inflammation; Chronic disease; Maternal Health; Obesity; Diabetes mellitus; Cardiovascular disease

I confirm that I have read this submission and believe that I have an appropriate level of expertise to confirm that it is of an acceptable scientific standard.

# Reviewer report: Reviewer response for version 1

**DOI:** 10.5256/f1000research.55161.r95825

**Published:** 2021-10-04

**Authors:** Tracy Reibel

**Affiliations:** 1 Telethon Kids Institute, Perth, WA, Australia; 2 Ngangk Yira Research Centre for Aboriginal Health and Social Equity, Murdoch University, Perth, WA, Australia

This is an important topic, as noted by the authors, there is insufficient evidence from well-designed studies to appropriately inform women of harms that may be associated with cannabis use in pregnancy. While the impact of alcohol on the developing fetus is now well established, this is not the case with cannabis use. It is broadly acknowledged that cannabis use is rising in many developed countries, as the legislation legalising use increases in many jurisdictions.

 While the survey response rate was small given the mailout (although anticipated in light of similar returns in pregnancy related surveys) it does raise questions regarding generalisability. This is noted by the authors in the discussion. My view is that the limitations could be made more explicit, by stating that there are a number of limitations from the study - all of which are described, just not named as such.

 My view also is that with just under half of the respondents disclosing cannabis use prior to pregnancy, this is a finding worthy of a stronger statement. It is highly likely that the sample was influenced by the participant's educational status, but nonetheless, an outcome that indicates that pregnant women may not only be prepared to participate in future studies on this topic but also honestly disclose cannabis use is worth stating. Especially when combined with a willingness to provide at least some biological samples.

 Overall, I found the manuscript to be well written, the methods and analysis were appropriately described, the discussion was a good reflection of the analysis. The conclusion that research is needed on this particular topic is long overdue and will eventually be an important addition to understanding the role of exposures on developing babies. This research has to be done though with sensitivity and assurance for women to be able to fully participate in future research.

Is the work clearly and accurately presented and does it cite the current literature?

Yes

If applicable, is the statistical analysis and its interpretation appropriate?

Yes

Are all the source data underlying the results available to ensure full reproducibility?

Yes

Is the study design appropriate and is the work technically sound?

Yes

Are the conclusions drawn adequately supported by the results?

Yes

Are sufficient details of methods and analysis provided to allow replication by others?

Yes

Reviewer Expertise:

Maternal and infant health, maternity care health systems, Aboriginal women, Fetal Alcohol Spectrum Disorder, women's health

I confirm that I have read this submission and believe that I have an appropriate level of expertise to confirm that it is of an acceptable scientific standard.
